# Association of body perception and dietary weight management behaviours among children and adolescents aged 6–17 years in China: cross-sectional study using CHNS (2015)

**DOI:** 10.1186/s12889-022-12574-6

**Published:** 2022-01-26

**Authors:** Lingling Song, Yong Zhang, Ting Chen, Patimaihan Maitusong, Xuemei Lian

**Affiliations:** 1 Department of Nutrition and Food Hygiene, School of Public Health and Management, Chongqing Medical University, Chongqing, 400016 China; 2 Chongqing Center for Disease Control and Prevention, Chongqing, 400042 China; 3 Center for Lipid Research, Key Laboratory of Molecular Biology for Infectious Diseases (Ministry of Education), Chongqing, 400016 China

**Keywords:** Dietary behaviours, Overweight, Obesity, Body perception, Body image dissatisfaction, Weight management behaviours

## Abstract

**Background:**

Obesity is rapidly globally increasing. Meanwhile, there is an increase in negative perception of the body image and, consequently, an increase in weight control management. Body perception plays an important mediating role between overweight or obesity and weight control behaviours. This study aims to examine whether body perception is associated with dietary weight management behaviours among Chinese children and adolescents.

**Methods:**

Child body perception and health behaviours from Chinese Health and Nutrition Survey (2015) were assessed, and 729 boys and 640 girls who aged 6 to 17 years were included in this study. Variables assessed as covariates were sociodemographic, physical activity and body perception involving self-perceptive weight status and body image dissatisfaction (BID). Multivariate logistic regression was used to explore the association between dietary weight management behaviours and body perception.

**Results:**

60.77% students rightly matched their body mass index (BMI) with self-perceived weight. The prevalence of BID was 59.39%. After adjustment for sociodemographic information, physical activities and BMI, those whose self-perceived weight status as overweight/obesity were associated with an increased risk of dietary weight management behaviours comparing to those who have normal weight in boys (OR = 5.07; 95% CI = 1.86, 13.87; *P* < 0.001) and girls (OR = 14.28; 95%CI = 5.73, 35.56; *P* < 0.001). And those who desire to be thinner were strongly associated with dietary weight management behaviours (OR = 9.51; 95%CI = 3.47, 26.09; *P* < 0.001) comparing to those who were satisfied with their body image in girls.

**Conclusions:**

The results revealed that body perception had a significant association with dietary weight management behaviours and exited the discrepancy according to sex. It is necessary to promote healthy awareness of body perception and to establish self-motivation for improving basic health knowledge in school health education activities.

## Background

Obesity is rapidly increasing worldwide and, particularly, in children and adolescents of developing countries [1]. From 1975 to 2016, the worldwide prevalence of obesity or overweight children and adolescents aged 5–19 years were reportedly rapidly increased more than four-fold from 4 to 18% globally [2]. Meanwhile, there is an increase in negative perception of the body image and, consequently, an increase in weight control management [3]. Body perception plays an important mediating role between overweight or obesity and weight control behaviours and has also been frequently reported in recent years in different population, particularly in female and in psychosocial domains [4–6].

Body perception is a complex feeling involving self-perception on weight and body shape, surrounded by the sensations and immediate experiences, also involving a subjective component that refers to individual satisfaction with body size and weight [7]. Body image dissatisfaction (BID) means the discrepancy between ideal body size and current body size. Previous studies have shown that body dissatisfaction, or the negative subjective evaluations/experiences of one’s physical appearance, including body size and shape [8], may increase the risk of unhealthy behaviours such as eating disorder or depression [9, 10]. Incorrect recognition of body weight status or body dissatisfaction image is a threat to weight control as it may be associated with unhealthy behaviours and psychosocial morbidities [11]. Hence, a correct self-perception of body may affect body weight management and, eventually, quality of life. Moreover, body perception could be a helpful tool for health care providers when a body weight management program was planned.

Although the specific route is not clear, it has been suggested that body perception may possess both direct and indirect associations with BMI, and dietary weight management behaviours. From one aspect, individual with a higher BMI increase the risk of BID and, subsequently, are more likely to adopt dietary management behaviours [12]. Additionally, Tang et al. [13] reported that BID is a mediator between body weight status and dietary weight management behaviours.

In recent years, the prevalence of overweight/obesity of children and adolescents has generally been increased in China. More and more pupils begun to pay attention to their body shape because of the “thinness is beauty” of mainstream under media’s coverage [14]. Meanwhile, pupils are prone to take misunderstandings when making their own body shape judgments due to the lack of health awareness, and that might lead to unhealthy weight control behaviours and psychosocial morbidities [11]. Even at these younger ages, body image is still an important part of the self-concept and body image dissatisfaction might be detrimental. Previous studies [12, 15] indicated that disturbances of body image and eating behaviour, which were formerly characterized as problems of adolescence, could originate before puberty.

Increasing prevalence of negative perception of the body image was reported in Chinese children, with more rapid increases of taking weight management behaviours in adolescents, as well as in girls versus boys [16, 17]. However, few studies have been conducted nationwide involving pupils and incorporating both body image dissatisfaction and self-perceived weight status together in terms of body perception. Therefore, in this study, our objectives were to identify the status of body perception and the association between body perception (included both self-perceptive weight status and BID) and dietary weight management behaviours among children and adolescents aged 6 to 17 years in China.

## Methods

### Study design and study population

This current cross-sectional study employed a secondary-analyses of data from the China Health and Nutrition Survey (CHNS) in 2015. CHNS was an open prospective cohort study that collected 10 waves of measurements on geography, economic development, public resources, and health indicators in China between 1989 and 2015 [18]. CHNS selected samples from 8 provinces in China by using multistage-random cluster process since 1989. Gradually, 15 provinces and municipalities were involved in the CHNS 2015. Counties and cities were stratified by income (low, middle and high) and a weighted sampling scheme was used to randomly select four counties and two cities in each province or municipality. Villages and townships within the counties, urban and suburban neighbourhoods within the cities were selected randomly. In each community, 20 households were randomly selected and all household members were interviewed. CHNS was approved by the Institutional Review Committees of the University of North Carolina at Chapel Hill and the National Institute of Nutrition and Food Safety, Chinese Center for Disease Control and Prevention [19].

All children and adolescents aged 6–17 years in households which attended the CHNS were interviewed face-to-face by trained interviewers at the participants’ home. Children under the age of 10 years completed the questionnaire with the assistance of their mothers or other key healthcare providers [18]. In this study, data related to children body perception and health behaviours from CHNS (2015) were assessed. We firstly included those who aged 6–17 years (*n* = 1752) and further excluded those whose data on self-perceptive weight status, BID or “whether on a diet” last year were missing (*n* = 215) and those whose data on other categorical variables were missing (*n* = 168). The final analyses included 1369 participants.

## Assessment of body perception

### Actual weight status

Using a body composition monitor scale BC601 (TANITA, Tokyo, Japan) or a portable wall-mounted metal tape SECA206 (SECA, Hangzhou, China) respectively, participants' weight and height were measured to the nearest 0.1 kg and 0.1 m in light indoor clothing and without shoes by trained and certified staff during the detailed physical examination [20]. BMI (kg/m^2^) was calculated by dividing weight (kg) by the height squared (m).

For data analysis, BMI was classified by underweight, normal and overweight/obesity. Obesity and overweight were defined using the Working Group on Obesity in China (WGOC) age-sex-specific BMI cut-offs [21]. Underweight was defined as BMI less than cut-off of age-sex-specific percentiles based on the “Screening Standard for Malnutrition of school age children and adolescents (WS/T456-2014)” [22]. Normal was defined as BMI less than cut-off of age-sex-specific overweight and more than cut-off of age-sex-specific underweight.

### Self-perceptive weight status

All children and adolescents were required to answer the question as “what do you think of your current weight status: underweight, normal or overweight/obesity”.

### Body image dissatisfaction

All participants were required to evaluate their figure by using the Figure Rating Scale (FRS). The scale consists of 9 contours, ranging from very thin (assignment of 1) to very obesity (assignment of 9) [23]. Studies have shown that a valid measurement of body satisfaction could be generated with this scale even in younger children [24, 25]. Participants were asked what they thought of their current silhouette or "how do you look like" (self-perceived body image) and which one they wanted their body to look like (ideal body image) by choosing a score [26], respectively.

BID variable was obtained by subtracting the participant’s ideal body FRS score from the present body FRS score. BID score ≥ 1 means that the participant “desired to be thinner”; BID score ≤ -1 demonstrates that the participant “desired to be heavier”; a BID score of zero means that the participant was satisfied with his or her figure [27].

### Ascertainment of dietary weight management behaviours

All children and adolescents were asked to report if they had tried to manage their weight through diet during the previous 12 months as follows: “no, I did not”, “yes, tried to lose weight” and “yes, tried to gain weight”.

### Covariates

We collected information on age, education level (elementary school and below, junior high school, high school and above), residence (urban, rural), parents’ education level (elementary school and below, junior high school, high school and above), sex (boys, girls), physical activities (no, regularly), and per capita household income (low, middle, high) through CHNS (2015) questionnaires.

### Statistical analysis

The skewness and kurtosis test for normality were performed on continuous variables, and the data were normally distributed if the p-values of both skewness and kurtosis test were greater than 0.05, otherwise, the data were skewed. Continuous variables of normal distribution are presented as mean ± standard deviation, such as age and BMI. No skewness distribution data observed in this study. Moreover, categorical variable such as sex, education level, residence, per capita household income, parental education level, and physical activity were described by frequency and percentage. The chi-square tests were used to compare the sex differences for categorical variables and the t-test was used to compare the difference in continuous data. If the expected value of a data grid is less than 5, we used the Fisher exact probability method for the test.

Consistency between participants’ actual weight status and self-perceptive weight status was assessed by the Kappa test. Weighted Kappa coefficients (criterion validity) were interpreted according to Landis and Koch for strength of agreement: Kappa < 0.20: poor agreement; Kappa = 0.21–0.40: fair agreement; Kappa = 0.41–0.60: moderate agreement; Kappa = 0.61–0.80: good agreement; and Kappa = 0.81–100: perfect/very good [28].

Multivariate logistic regression analyses were performed to determine the association of the following outcomes behaviours with body perception stratifying by sex: dietary weight management (0 = no behaviours, 1 = behaviours). Multivariable model 1 was adjusted for age (continuous). Multivariable model 2 was further adjusted for residence (0 = urban, 1 = rural), per capita household income (0 = low level, 1 = middle level, 2 = high level), education level (0 = elementary school and below, 1 = junior high school, 2 = high school and above), mother’s education level (0 = elementary school and below, 1 = junior high school, 2 = high school and above), father’s education level (0 = elementary school and below, 1 = junior high school, 2 = high school and above), and physical activity (0 = no, 1 = regular). The model 3 was further adjusted for BMI (0 = normal, 1 = underweight, 2 = overweight/obesity).

All statistical analyses were performed by using STATA 15.0 (StataCorp., College Station, TX). Statistical significance was set as *P* < 0.05 and all statistical tests of hypothesis were two sided. All methods were performed in accordance with the relevant guidelines and regulations.

## Results

### Sample characteristics

This study included 1369 children and adolescents that aged 6–17 years from urban (33.97%) and rural (66.03%) areas in China. Boys accounted for 53.25% and girls accounted for 46.75% among the participants. The mean age was 10.37 years (10.38 and 10.37 years for boys and girls, respectively). Most of the participants were elementary school students and below (68.81%). Compared with girls, boys have higher BMI (*P* < 0.05). There was no sex difference in distributions of other sociodemographic variables and physical activity (Table 1).

**Table 1:** Characteristics of Children and Adolescents According to Sex in the CHNS (2015)

Characteristics	Total (*N* = 1,369)	Boys (*N* = 729)	Girls (*N* = 640)	*P*
**BMI, kg/m**^**2**^	17.86 ± 3.31	18.08 ± 3.39	17.61 ± 3.20	**0.008**
**Age, years**	10.37 ± 3.12	10.38 ± 3.18	10.37 ± 3.06	0.932
**Grade**^**a**^				0.491
Elementary school and below	942 (68.81)	499 (68.45)	443 (69.22)	
Junior high school	312 (22.79)	174 (23.87)	138 (21.56)	
High school and above	109 (7.96)	54 (7.41)	55 (8.59)	
**Residence**				0.119
Urban	465 (33.97)	234 (32.10)	231 (36.09)	
Rural	904 (66.03)	495 (67.90)	409 (63.91)	
**Per capita household income**				0.203
Low income	447 (32.65)	225 (30.86)	222 (34.69)	
Middle income	456 (33.31)	242 (33.20)	214 (33.44)	
High income	466 (34.04)	262 (35.94)	204 (31.87)	
**Father’s education level**^**b**^				0.510
Elementary school and below	132 (9.64)	66 (9.10)	66 (10.31)	
Junior high school	433 (31.63)	241 (33.10)	192 (30.00)	
High school and above	436 (31.85)	234 (32.10)	202 (31.56)	
**Mother’s education level**^**c**^				0.622
Elementary school and below	196 (14.32)	98 (13.44)	98 (15.31)	
Junior high school	466 (34.04)	251 (34.43)	215 (33.59)	
High school and above	405 (29.58)	209 (28.67)	196 (30.63)	
**Physical activity**^**d**^				0.126
No	830 (60.63)	427 (58.57)	403 (62.97)	
Regular	526 (38.42)	293 (40.19)	233 (36.41)	

*Note:* The chi-square tests were used to examine significant sex difference in terms of grade, parental education level, residential area, per capita household income and physical activity. The t-test was used to examine significant sex difference in terms of age and BMI. Value is mean ± SD or N (%). Proportions are column percentages. ^a,b,c,d^ few data missing (grade 6; father’s education level 368; mother’s education level 302; physical activity 13). Bold values represent statistically significant (*P* < 0.05)

### Overweight/obesity prevalence and body perception

The finding showed that the prevalence of overweight/obesity was 26.95%. However, only 12.09% participants self-perceived weight status as overweight/obesity. Additionally, the majority of participants (71.76%) self-assessed their weight as normal status. The prevalence of children and adolescents dissatisfied with their body image was 59.39% (Table 2). Table 2 also presents that, regarding to the sample’s characteristics, there have differences in the distribution of BMI among different sex, mothers’ education levels, fathers’ education levels, and per capita household income levels. Besides, we found difference of distribution of self-perceptive weight status which were manifested in residential, parental education levels, and per capita household income levels. Furthermore, the differences in the distribution of BID in grade, residence, parents’ education level, and per capita household income were statistically significant.

**Table 2:** Comparison of distribution of BMI, self-perceptive weight and BID of children and adolescents aged 6–17 years with different sociological characteristics (*N* = 1,369, (%))

Characteristics		BMI			*P*	Self-perceptive weight			*P*	BID			*P*
		Normal	Underweight	Overweight/Obesity		Normal	Underweight	Overweight/Obesity		Satisfied	Desire to be heavier	Desire to be thinner	
**Sex**	Boys	467(64.06)	53(7.27)	209(28.67)	0.160	520(71.33)	119(16.32)	90(12.35)	0.826	292(40.05)	266(36.49)	171(23.46)	0.317
	Girls	420(65.63)	60(9.38)	160(25.00)		466(72.81)	100(15.63)	74(11.56)		264(41.25)	210(32.81)	166(25.94)	
**Grade**^**a**^	Elementary school	557(59.13)	85(9.02)	300(31.85)	**0.000**	681(72.29)	159(16.88)	102(10.83)	0.129	366(38.85)	356(37.79)	220(23.35)	**0.003**
	Middle school	235(75.32)	24(7.69)	53(16.99)		223(71.47)	47(15.06)	42(13.46)		135(43.27)	93(29.81)	84(26.92)	
	Junior school	90(82.57)	4(3.67)	15(13.76)		76(69.72)	13(11.93)	20(18.35)		54(49.54)	23(21.10)	32(29.36)	
**Residence**	Urban	301(64.73)	28(6.02)	136(29.25)	0.065	333(71.61)	62(13.33)	70(15.05)	**0.013**	223(47.96)	120(25.81)	122(26.24)	**0.000**
	Rural	586(64.82)	85(9.40)	233(25.77)		653(72.23)	157(17.37)	94(10.40)		333(36.84)	356(39.38)	215(23.78)	
**Mother’s education level**^**b**^	Elementary school	131(66.84)	20(10.20)	45(22.96)	**0.007**	142(72.45)	38(19.39)	16(8.16)	**0.008**	75(38.27)	71(36.22)	50(25.51)	**0.001**
	Middle school	296(63.52)	47(10.09)	123(26.39)		327(70.17)	82(17.60)	57(12.23)		175(37.55)	191(40.99)	100(21.46)	
	Junior school	256(63.21)	19(4.69)	130(32.10)		285(70.37)	51(12.59)	69(17.04)		182(44.94)	109(26.91)	114(28.15)	
**Father’s** **education level**^**c**^	Elementary school	88(66.67)	19(14.39)	25(18.94)	**0.000**	85(64.39)	36(27.27)	11(8.33)	**0.000**	47(35.61)	51(38.64)	34(25.76)	**0.040**
	Middle school	278(64.20)	48(11.09)	107(24.71)		308(71.13)	77(17.78)	48(11.09)		169(39.03)	171(39.49)	93(21.48)	
	Junior school	266(61.01)	20(4.59)	150(34.40)		318(72.94)	44(10.09)	74(16.97)		186(42.66)	132(30.28)	118(27.06)	
**Per capita household income**	Low	302(67.56)	50(11.19)	95(21.25)	**0.000**	318(71.14)	92(20.58)	37(8.28)	**0.000**	174(38.93)	178(39.82)	95(21.25)	**0.012**
	Middle	303(66.45)	40(8.77)	113(24.78)		322(70.61)	79(17.32)	55(12.06)		186(40.79)	161(35.31)	109(23.90)	
	High	282(60.52)	23(4.94)	161(34.55)		346(74.25)	48(10.30)	72(15.45)		196(42.06)	137(29.40)	133(28.54)	
**Physical activity**^**d**^	No	531(63.98)	79(9.52)	220(26.51)	0.137	585(70.48)	144(17.35)	101(12.17)	0.302	333(40.12)	294(35.42)	203(24.46)	0.627
	Regular	345(65.59)	34(6.46)	147(27.95)		388(73.76)	75(14.26)	63(11.98)		221(42.02)	173(32.89)	132(25.10)	
**Total**		887(64.79)	113(8.26)	369(26.95)		986(71.76)	219(16.15)	164(12.09)		556(40.61)	476(34.77)	337(24.62)	

*Note*: The chi-square tests were used to examine difference of distribution of BMI self-perceived weight and BID in terms of sex, grade, parental education level, residential area, per capita household income and physical activity. Value is N (%). Proportions are row percentages. ^a,b,c,d^ few data missing (grade 6; father’s educational level 368; mother’s educational level 302; physical activity 13)

### Agreement between self-perceptive weight status and measured BMI categories

**Fig. 1:**
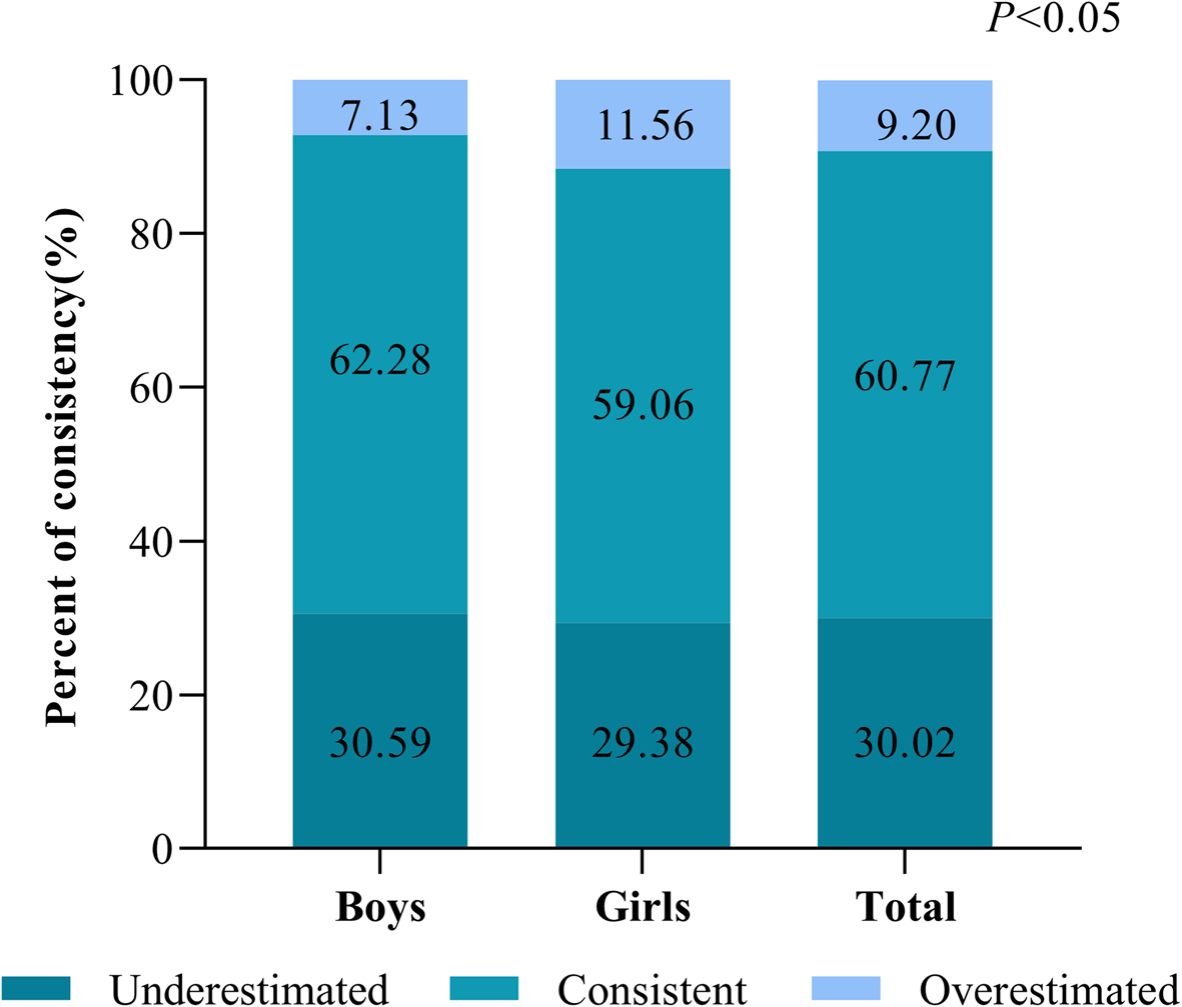
Consistency of self-perceived weight status and actual weight status (BMI) according to sex among children and adolescents aged 6 to 17 years in the CHNS, 2015 (*N* = 1,369). The chi-square tests were used to examine agreement between self-perceived weight status and actual weight status. If the participants self-perceived weight status corresponded with their weight status, they were classified into the consistent assessment group. If the participants self-perceived weight status was below their weight status, they were classified into the underestimated group. Otherwise, they were classified into the overestimated group

Approximately 60% students rightly matched their BMI category with self-perceived weight status (Fig. 1). Girls prefer to over-estimated their weight status compared to boys (11.56% vs 7.13%). Additionally, boys perceived more accurately their own weight status (62.28% vs 59.06%, *P* < 0.05). There was a significant, strong discrepancy between self-perceptive weight status and actual weight status (*P* < 0.05), as shown in Table 3. The level of agreement between self-perceptive weight status and actual weight status was poor (Kappa = 0.1960) for the total sample, and boys' Kappa values (Kappa = 0.2391) were higher than girls' (Kappa = 0.1447, Table 3).

**Table 3:** Comparing self-perceptive weight and actual weight status of children and adolescents aged 6–17 years in China using Kappa test and Chi-square test (*N* = 1,369, (%))

**Self-perceptive weight status**	**Actual Weight Status**			**Kappa**	**χ**^**2**^	***P***
	**Underweight**	**Normal**	**Overweight/Obesity**			
**Boys**						
Underweight	24 (45.28)	80 (17.13)	15 (7.18)	0.2391	134.664	**0.000**
Normal	28 (52.83)	364 (77.94)	128 (61.24)			
Overweight/Obesity	1 (1.89)	23 (4.93)	66 (31.58)			
**Girls**						
Underweight	26 (43.33)	61 (14.52)	13 (8.13)	0.1447	56.761	**0.000**
Normal	33 (55.00)		114 (71.25)			
Overweight/Obesity	1 (1.67)	40 (9.52)	33 (20.63)			
**Total**						
Underweight	50 (44.25)	141 (15.90)	28 (7.59)	0.1960	178.343	**0.000**
Normal	61 (53.98)	683 (77.00)	242 (65.58)			
Overweight/Obesity	2 (1.77)	63 (7.10)	99 (26.83)			

*Note:* The chi-square tests and the Kappa test were used to examine agreement between self-perceived weight status and actual weight status. Value is N (%). Proportions are column percentages. Bold values represent statistically significant (*P* < 0.05)

### Association between body perception and dietary weight management behaviours

In the study, we documented 93 incident of dietary weight management behaviours, including 40 cases for gaining weight and 53 cases for losing weight. The difference of distribution of dietary weight management behaviours by BMI, self-perceptive weight status and BID were statistically significant (Table 4).

**Table 4:** Distribution of dietary weight management behaviours by BMI, Self-perceptive weight and BID among Chinese children and adolescents aged 6–17 years (*N* = 1,369, (%))

Variables	Dietary weight management behaviours			χ^2^	*P*
	**No**	**Yes, tried to gain weight**	**Yes, tried to lose weight**		
**BMI**					
Normal	838(94.48)	24(2.71)	25(2.82)	19.4347	**0.001**
Underweight	106(93.81)	6(5.31)	1(0.88)		
Overweight/obesity	332(89.97)	10(2.71)	27(7.32)		
**Self-perceptive weight**					
Normal	952(96.55)	16(1.62)	18(1.83)	168.2609	**0.000**
Underweight	206(94.06)	12(5.48)	1(0.46)		
Overweight/obesity	118(71.95)	12(7.32)	34(20.73)		
**BID**					
Satisfied	534(96.04)	10(1.80)	12(2.16)	65.8675	**0.000**
Desire to be heavier	458(96.22)	13(2.73)	5(1.05)		
Desire to be thinner	284(84.27)	17(5.04)	36(10.68)		

*Note:* The chi-square tests were used to examine dietary weight management behaviours difference in terms of BMI, BID and self-perceived weight status. Value is N (%). Proportions are row percentages. Bold values represent statistically significant (*P* < 0.05). BMI = body mass index; BID = body image dissatisfaction

After adjusting for age (Model 1), overweight/obesity of self-perceptive weight status were significantly associated with a higher risk to take behaviours for managing their weight compared with self-perceptive weight status as normal in boys [OR (95%CI) = 7.86(3.90, 15.87)] and girls [OR (95%CI) = 13.18(6.58, 26.41)]. In boys, the associations were slightly attenuated but remained significant after further adjusting for sociodemographic characteristics including residence, grade, per capita household income, parent education level, and physical activity (Model 2) in boys [OR (95%CI) = 6.01(2.54, 14.20)]. The same was true after adjusting the BMI (Model 3) [OR (95%CI) = 5.07(1.86, 13.87)]. Meanwhile, in girls, the correlations were slightly increased after further adjusted for sociodemographic characteristics (Model 2) [OR (95%CI) = 13.93(5.86, 33.11)] and BMI (Model 3) [OR (95%CI) = 14.28(5.73, 35.56)] (Table 5).

**Table 5:** Association between body perception and dietary weight management behaviours in children adolescents stratified by sex in CHNS (2015)

Variables			Model 1	Model 2	Model 3
			OR (95% CI)	OR (95% CI)	OR (95% CI)
**Boys**	**Self-perceptive weight**	Normal	Ref	Ref	Ref
		Underweight	2.15(0.90, 5.12)	1.85(0.61, 5.61)	1.98(0.64, 6.12)
		Overweight/obesity	**7.86(3.90, 15.87)**^**b**^	**6.01(2.54, 14.20)**^**b**^	**5.07(1.86, 13.87)**^**b**^
	**BID**	Satisfied	Ref	Ref	Ref
		Desire to be heavier	0.97(0.42, 2.21)	0.85(0.32, 2.23)	0.92(0.34, 2.47)
		Desire to be thinner	**2.86(1.38, 5.92)**^**a**^	1.80(0.73, 4.42)	1.47(0.58, 3.72)
**Girls**	**Self-perceptive weight**	Normal	Ref	Ref	Ref
		Underweight	1.43(0.51, 3.97)	2.22(0.65, 7.61)	1.67(0.44, 6.34)
		Overweight/obesity	**13.18(6.58, 26.41)**^**b**^	**13.93(5.86, 33.11)**^**b**^	**14.28(5.73, 35.56)**^**b**^
	**BID**	Satisfied	Ref	Ref	Ref
		Desire to be heavier	1.02(0.37, 2.80)	2.02(0.60, 6.76)	1.88(0.54, 6.48)
		Desire to be thinner	**6.51(3.01, 14.07)**^**b**^	**8.97(3.38, 23.80)**^**b**^	**9.51(3.47, 26.09)**^**b**^

*Note:* Multiple logistic regression models were used in this analysis. The model 1 was adjusted for age (years). The model 2 was further adjusted for residence (urban, rural), per capita household income (lower, middle, higher), grade (elementary, junior, high), mother’s education level (elementary, junior, high), father’s education level (elementary, junior, high), and physical activity (no, regular). The model 3 was further adjusted for BMI (normal, underweight, overweight/ obesity). Significant between-group differences were shown in bold. ^a^*P* < 0.01, ^b^*P* < 0.001

As expected, after adjusting for age, those who desire to be thinner significantly associated with a higher risk to take behaviours for managing their weight compared with those who were satisfied with their body in girls [OR (95%CI) = 6.51(3.01, 14.07)]. The change of associations between BID and dietary weight management behaviours were similar to that of self-estimated weight status after adjusting for sociodemographic characteristics [OR (95%CI) = 8.97(3.38, 23.80)] and BMI [OR (95%CI) = 9.51(3.47, 26.09)] in girls (Table 5).

## Discussion

This study aims to evaluate the body perception and its association with dietary weight management behaviours among Chinese children and adolescents aged 6 to 17 years. We found that the children and adolescents’ body perception showed a significant association with their dietary weight management behaviours.

The finding showed that the prevalence of overweight/obesity was 26.95% and underweight was 8.26% in China (Table 2). Similarly, Zheng et al. reported the prevalence of overweight/obesity of children and adolescents was 29.8% using data from five major cities (Beijing, Shanghai, Nanjing, Xian, and Chengdu) across China [29]. Interestingly, we observed that around two fifths (39.23%) of the total sample were misperception on their weight status (Fig. 1) and over half (59.39%) of the total sample were dissatisfied with their body contour (Table 2). This is consistent with previous study result among Chinese children and adolescents [30]. Similar results regarding weight perception on other countries, a study showed a high prevalence of weight status misperception (40%) among Korean high school students [31] and Brazilian study displayed 34% prevalence of misperceiving weight among adolescents aged 12–17 years [32].

As another important finding from our study, there is a discrepancy in body perception (self-perceptive weight status or BID) according to sex. Boys prefer to underestimate their weight status but girls more likely to overestimate in our study. The results were consistent with the studies of Wang VH et al. [33] and Xie B et al. [34]. The reason may be the media promotes thinness and beauty. Media pressure was proven to increase the likelihood of appearance anxiety and body shame in public, particularly. Girls are more likely to increase feeling body shame and appearance anxiety after viewing advertisements featuring idealized images [35]. The degree of negative emotions (such as shame, guilt) that girls are exposed to psychological stress which is generated by the self-perceived versus desired body image discrepancy is heavier than that of boys. Hence, females tend to be more sensitive towards their weight status and body image duo to external pressure (media, family, or peer) and internal pressure than their male counterparts [36]. It was also interesting to find that boys prefer to be heavier comparing to girls in China, which was consistent with other studies [37] demonstrating that frequent exposure to the robust bodies of media models arouse a significant threat to young men BID. These results could also be explained by Chinese traditional culture that parents and grandparents have a stereotypical image of children—chubby boys and slim girls.

Back to the topic of dietary weight management behaviours, no matter boys or girls, participants whose self-perceptive weight status were overweight/obesity were most likely to have dietary weight management behaviours comparing to those whose self-perceptive weight status were normal (Table 5), which has also been confirmed in other studies [38]. De Guzman et al. [39] found that higher dissatisfaction of body image during puberty may play an important role for increasing prevalence of dietary weight management behaviours. Another longitudinal study [40] has indicted that being labelled as fat or having a negative body image perception was associated with heavier weight gain. In this study, the degree of association between the dietary weight management behaviours and body perception has a discrepancy in sex after adjusting BMI (Table 5). The reason might be the trait of an individual with a higher BMI may be more prone to feel body dissatisfaction and body image inflexibility due to weight stigma and health inequities. Body dissatisfaction appears to contribute to an individual feeling reluctant to experience their negative body image, and thus contributing to an increased likelihood of engaging in disordered eating behaviours (dietary restraint or crapulent behaviour), which is particularly pronounced among women [7]. Additionally, girls are less able to withstand external pressure, especially discrepancy between ideal and actual appearance, making it easier to adopt management weight behaviours through eating due to negative emotional backlogs in comparison to boys [41].

The dissatisfaction with one’s current body image or weight fosters weight management behaviours and related cognitions in order to change one’s appearance [42]. A series of published literature shows [12, 32, 43] that weight management behaviours may be affected by subjective factors like self-perceived weight and body image perception and objective factors included parental education level, peer pressure, social culture, social media, and psychologic etc. Hence, schools should promote health education to improve awareness of self-motivation and to encourage students possess healthy dietary behaviours and proper weight perceptions.

This study has several limitations that need to be acknowledged. First, this study involved children and adolescents age 6 to 17 years old, but most of them are primary school students (68.81%). Hence, there were only 93 out of 1,369 study participants reported that they had weight management behaviours including gaining weight or losing weight in the past year. Therefore, the results may include a selection bias due to uneven distribution of grades, and might not represent the entire Chinese children and adolescent population. Moreover, as the data used in the current study was cross sectional, the findings were correlations, not causations, with the inability to decide the direction of the effects, thus future research should use a longitudinal design or experiments to examine how body perception interact with dietary weight management behaviours over time.

## Conclusions

In this study, the prevalence of overweight/obesity among Chinese children and adolescents aged 6 to 17 years was 26.95%. The accuracy of weight perception and body image satisfaction is not optimistic among Chinese children and adolescents. Body perception showed a significant association with dietary weight management behaviours. It is necessary to promote healthy body image perceptions and establish self-motivation for improving basic health knowledge in school health education activities.

## Data Availability

Data is available on request to official website of the China Health and Nutrition Survey, China. (https://www.cpc.unc.edu/projects/china/data/datasets.)

## Abbreviations

BID: Body image dissatisfaction
BMI: Body mass index
CHNS: China Health and Nutrition Survey
CI: Confidence interval
OR: Odds ratio
Ref: Reference
